# ECLS supported transport of ICU patients: does out-of -house implantation impact survival?

**DOI:** 10.1186/s13019-021-01508-9

**Published:** 2021-06-02

**Authors:** Felix Fleissner, Alexandru Mogaldea, Andreas Martens, Ruslan Natanov, Stefan Rümke, Jawad Salman, Tim Kaufeld, Fabio Ius, Erik Beckmann, Axel Haverich, Christian Kühn

**Affiliations:** Department of Cardiothoracic, Transplant and Vascular Surgery, Hannover Medical School, Carl-Neuberg Strasse 1, 30625 Hannover, Germany

**Keywords:** ECLS, Transport, ARDS, Cardiogenic shock

## Abstract

**Background:**

Extracorporeal life support (ECLS) is an established tool to stabilize severely ill patients with therapy-refractory hemodynamic or respiratory failure. Recently, we established a mobile ECLS retrieval service at our institution. However, data on the outcome of patients receiving ECLS at outside hospitals for transportation into tertiary hospitals is still sparse.

**Methods:**

We have analyzed all patients receiving ECLS in outside hospitals (Transport group, TG) prior to transportation to our institution and compared the outcome to our in-house ECLS experience (Home Group, HG).

**Results:**

Between 2012 and 2018, we performed 978 ECLS implantations, 243 of which were performed on-site in tertiary hospitals for ECLS supported transportation. Significantly more veno-venous systems were implanted in TG (*n* = 129 (53%) vs. *n* = 327 (45%), *p* = 0.012). Indication for ECLS support differed between the groups, with more pneumonia; acute respiratory distress syndromes in the TG group and of course, more postcardiotomy patients in HG. Mean age was 47 (± 20) (HG) vs. 48 (± 18) (TG) years, *p* = 0.477 with no change over time. No differences were seen in ECLS support time (8.03 days ±8.19 days HG vs 7.81 days ±6.71 days TG, *p* = 0.675). 30-day mortality (*n* = 379 (52%) (HG) vs. *n* = 119 (49%) (TG) *p* = 0.265) and death on ECLS support (*n* = 322 (44%) (HG) vs. *n* = 97 (40%) TG, *p* = 0.162) were comparable between the two groups, despite a more severe SAVE score in the v-a TG (HG: − 1.56 (± 4.73) vs. TG -3.93 (± 4.22) *p* < 0.001). Mortality rates did not change significantly over the years. Multivariate risk analysis revealed Influenza, Peak Insp. Pressure at implantation, pO2/FiO2 ratio and ECLS Score (SAVE/RESP) as well as ECLS support time to be independent risk factors for mortality.

**Conclusion:**

Mobile ECLS support is a tremendous challenge. However, it is justified to offer 24 h/7d ECLS standby for secondary and primary hospitals as a tertiary hospital. Increasing indications and total numbers for ECLS support raise the need for further studies to evaluate outcome in these patients.

## Introduction

Extracorporeal life support (ECLS) is a potentially life-saving technique applied to critically ill patients with severe cardiac and/or pulmonary failure, and its use has increased over the past decades [1, 2].

Generally, two forms of ECLS are available: venous-venous (v-v) support for hypoxic respiratory failure and acute respiratory distress syndrome (ARDS), and venous-arterial support for cardiac failure/cardiogenic shock [3].

The use of ECLS is expanding, and with growing experience and availability of reliable devices, this treatment option is no longer limited to large, tertiary centers but is also available in some smaller, secondary centers. However, an increasing number of tertiary centers are now offering mobile ECLS teams to transport severely ill patients from smaller secondary and primary centers with the possibility of ECLS cannulation on-site. Since the first report of a successful ECLS transport in 1986, this potentially life-saving treatment option has gained widespread use [4]. In recent years, a number of single center studies have been published on the experience with mobile ECLS teams. Here, we present our experience with the mobile ECLS team and compare its outcome to our extensive in-house experience. We aim at answering the question whether extra-hospital ECLS implantation has an influence on mortality and morbidity.

## Methods

We retrospectively analyzed all extracorporeal membrane oxygenation implantations performed in-house at our center and at outside hospitals from 2012 until 2018.

During this time, a total of 978 ECLS implantations were performed, of which 243 were performed on-site in tertiary hospitals for ECLS supported transportation. Contact was usually initiated by the referring clinic to the cardiac surgery department by telephone. After careful consideration with the collaborating departments, the patient was selected for either v-v or v-a support. Patients eligible for v-v ECLS support were critically ill patients with potentially reversible respiratory failure or indication/candidate for lung transplant with a peak inspiratory pressure > 32 mmHg, refractory hypercarbia (pH < 7.2) despite optimal respiratory maneuvers. Indications for v-a support were patients in cardiogenic shock with treatment options (reversible/VAD/transplant/surgery candidate) with acedemia, lactatemia, under high doses of catecholamines.

The mobile ECMO team consists of a cardiac surgeon and a perfusionist. Transport was carried out as appropriate, either ground based or via helicopter/airplane for longer distances (> 150 km). We used the CARDIOHELP-System (Getinge, Getinge, Sweden) console and a variety of cannulas (Novaport, Avalon, BioMedicus, Twinport, HLS) at the surgeon’s discretion. Based on our experience with femoral v-a cannulation, we have a mandatory approach to establish distal limb perfusion. If not possible on-site, distal limb perfusion is performed either through open surgery or ultrasound-guided after arrival at our center. V-v support is either performed via cannulation of the femoral vein and the jugular vein or by a twin-port canula via the femoral vein if hypercarbia is the main reason for respiratory failure.

Patients characteristics differed significantly between the in-house group (HG) and the transport group (TG) (Table 1). Significantly more patients in the TG had respiratory failure (Pneumonia/ARDS) and Myocarditis, whereas more patients in the HG were post-cardiotomy, post-VAD or post-transplant (lung transplant and/or heart transplant) patients. Mean age was 47 (HG) vs. 48 (TG) years, *p* = 0.477 with no change over time. 66% of patients were male. SAVE score (for v-a support) was significantly lower in the TG (HG: − 1.56 (± 4.73) vs. TG -3.93 (± 4.22) *p* < 0.001), whereas RESP scores (for v-v support) were not significantly different between the two groups (HG -1.14 (± 4.146) vs. TG -0.52 (± 3.635) *p* = 0.130) (Table 2). Significantly more patients of the HG were on dialysis prior to ECLS implantation (HG *n* = 263 (36%) vs. TG *n* = 34 (14%) *p* < 0.001). Peak inspiratory pressure was higher in the HG compared to the TG (34 (± 6) 32 (+ − 8), *p* = 0.009).

**Table 1:** Indication for ECLS support comparing in house implantation (Home) vs. external implantations (Transport)

Indication for ECLS	all	Home	Transport	p
Total	978 (100)	733	243	
Pneumonia	292 (30)	189 (26)	102 (42)	***p*** **< 0.001**
Influenca A	48 (5)	30 (4)	18 (7)	*p* = 0.32
ARDS	425 (44)	286 (39)	138 (57)	***p*** **= < 0.001**
Lungfibrosis	33 (3)	30 (4)	3 (1)	***p*** **= 0.038**
Pulmonary hypertension	43 (4)	35 (5)	8 (3)	*p* = 0.216
Cystic fibrosis	29 (3)	25 (3)	4 (2)	*p* = 0.373
Lung embolia	50 (5)	39 (5)	11 (5)	*p* = 0.384
PMR/VSD/ASD	29 (3)	19 (3)	10 (4)	*p* = 0.160
Myocardial infarction (NSTEMI, STEMI)	118 (12)	83 (11)	35 (14)	*p* = 0.212
DCM	80 (8)	64 (9)	15 (6)	*p* = 0.224
Myocarditis	26 (3)	15 (2)	11 (5)	***p*** **= 0.062**
Peripartum cardiomyopathy	5 (0.5)	2 (0.3)	3 (1)	*p* = 0.224
Polytrauma	21 (2)	13 (2)	8 (3)	*p* = 0.102
Sepsis	49 (5)	49 (7)	0	***p*** **< 0.001**
post DLTX	30 (3)	30 (4)	0	***p*** **< 0.001**
Postcardiectomie	2 (0.2)	2 (0.3)	1 (0.4)	*p* = 0.564
Burn	16 (2)	16 (2)	0	*p* = 0.410
Post HTX	20 (2)	20 (3)	4 (2)	***p*** **= 0.006**
Cardiac failure after VAD	34 (4)	33 (5)	1 (0.4)	***p*** **= 0.001**
Postcardiotomie	109 (11)	108 (15)	1 (0.4)	***p*** **< 0.001**
Others	92 (9)	83 (11)	9 (4)	***p*** **< 0.001**

ARDS: acute respiratory distress syndrome, PMR: Papillary muscle rupture, VSD: ventricular septum defect, ASD: atrial septum defect

**Table 2:** Indication for ECLS support in the Transport group

Indication for ECMO (transport only)	total	v-v	v-a	p
Total	243	114	129	
Pneumonia	102 (42)	89	11	*p* < 0.001
Influenza A	18 (7)	16	2	*p* < 0.001
ARDS	138 (57)	118	16	*p* < 0.001
Lungfibrosis	3 (1)	2	1	*p* = 0.577
Pulmonary hypertension	8 (3)	2	6	*p* = 0.84
Cystic fibrosis	4 (2)	4	0	*p* = 0.091
Lung embolia	11 (5)	0	11	*p* < 0.001
PMR/VSD/ASD	10 (4)	2	8	*p* = 0.024
Myocardial infarction (NSTEMI, STEMI)	35 (14)	1	34	*p* < 0.001
DCM	15 (6)	0	14	*p* < 0.001
Myocarditis	11 (5)	0	11	*p* < 0.001
Peripartum cardiomyopathy	3 (1)	0	3	*p* = 0.089
Polytrauma	8 (3)	8	0	*p* = 0.008
Sepsis	0	0	0	
Post DLTX	0	0	0	
Postcardiectomie	1 (0.4)	0	0	*p* = 0.285
Burn	0		0	*p* = 0.362
post HTX	4 (2)	0	0	
Cardiac failure after VAD	1 (0.4)	0	1	*p* = 0.449
Postcardiotomie	1 (0.4)	0	1	*P* = 0.449
Others	9 (4)	6	3	*p* = 0.362

v-v: veno-venous support, v-a veno-arterial support, dltx: Double lung transplantation, DCM: dilative cardiomyopathy

### Statistical methods

Data analysis was performed using SPSS 24.0 (IBM, Armonk, NY, USA). Primary end-points were mortality and morbidity. Categorical and continuous variables were summarized as proportions (percentages), and mean (standard deviation) or median with 25–75% IQR, respectively. The independent-samples Student’s *t*-test and the *χ*^2^ test were used for comparisons of continuous and categorical variables among included and excluded patients, respectively.

Fisher’s exact test was used for analysis. Univariable analysis was performed to identify risk factors for study end-points. Multivariable analysis using a forward stepwise logistic regression model was performed to identify independent risk factors for study end-points. Results were reported as odds ratio (OR) with a 95% confidence interval (CI). Two-tailed *P*-values < 0.05 were considered significant.

## Results

Cannulation was successful in all patients except one who died during cannulation. All but one ECMO were implanted by our own team. In the TG, more v-v support was established (HG *n* = 327 (45%) vs. TG *n* = 129 (53%), *p* = 0.027) whereas in the HG, more patients received v-a support (HG *n* = 384 (52%) vs. TG *n* = 109 (45%), *p* = 0.027). A minority of patients needed additional cannulation due to combined respiratory and cardiac failure. Time to ECLS support after admission to hospital was not significantly different between the two groups (HG 46 h (± 41 h) vs. TG 52 h (± 89 h), *p* = 0.177). A significantly higher number of awake ECLS were implanted in the HG compared to TG (HG *n* = 144 (20%) vs. TG *n* = 16 (7%), *p* < 0.001). In TG, there were the obvious differences due to indication with v-a for cardiac failure and v-v for respiratory failure (Table 2). In 9% of the TG, subsequent changes in the support were made after transportation to our center (either switching from v-v to v-a or additional cannulas for v-a-v and v-v-a support).

There were no transportation-related complications and all patients survived the transport to our clinic after successful ECLS implantation.

ECLS support times were comparable between the two groups with (8.03 days ±8.19 days HG vs 7.81 days ±6.71 days TG, *p* = 0.675).

Rates for mortality and death on ECLS support were comparable between the two groups with *n* = 379 (52%) (HG) vs. *n* = 119 (49%) (TG) *p* = 0.265 and *n* = 322 (44%) (HG) vs. *n* = 97 (40%) TG, *p* = 0.162, respectively, despite a more severe SAVE score in the v-a TG (HG: − 1.56 (± 4.73) vs. TG -3.93 (± 4.22) *p* < 0.001). However, more patients in the TG suffered from death after successful ECLS weaning (HG: *n* = 52 (7%) vs. TG *n* = 32 (13%), *p* = 0.003). The vascular complication rate (all complications with necessity for surgical intervention) was comparable between both groups with HG *n* = 40 (5%) vs. TG *n* = 13 (5%), *p* = 0.5, respectively. As expected, v-a patients had a significantly higher incidence of vascular complications compared to the v-v group (*n* = 11 v-a group vs. *n* = 2 v-v group (TG only), *p* = 0.003).

Interestingly, there was no significant difference in the TG with regards to outcome after v-a and v-v support. Exchange of the oxygenator was necessary in only 1.2% of all patients with no significant difference between v-a and v-v systems (TG only) (see Table 3).

**Table 3:** Patients characteristic of the transport group

Patients characteristics	Home	Transport	p
Age (yrs)	47 (+ − 20)	48 (+ − 18)	*p* = 0.477
Time admission to circulatory support (h)	46 (+ − 41)	52 (+ − 89)	*p* = 0.177
Gender (male) n(%)	478 (65)	161 (66)	*p* = 0.756
Awake ECMO	144 (20)	16 (7)	***p*** **< 0.001**
Dialysis before ECMO n (%)	263 (36)	34 (14)	***p*** **< 0.001**
Peak Insp. Pressure, cmH2O	34 (+ − 6)	32 (+ − 8)	***p*** **= 0.009**
pO2/FiO2	96 (+ − 29)	93 (+ − 18)	*p* = 0.343
SAVE Score (v-a only)	−1.56 (+ − 4.73	−3.93 (+ − 4.22)	***p*** **< 0.001**
RESP Score (v-v only)	−1.14 (+ − 4.146)	−0.52(+ − 3.635)	*p* = 0.130
**type of support**	**Home**	**Transport**	**p**
Total n	733	243	
v-a n (%)	384 (52)	109 (45)	***p*** **= 0.027**
v-v n (%)	327 (45)	129 (53)	***p*** **= 0.012**
Others n (%)	22 (3)	5 (2)	*p* = 0.259
**perioperative data**	**Home**	**Transport**	**p**
ECLS running time (d)	8.03 (+ − 8.19)	7.81 (+ − 6.71)	*p* = 0.675
Mortality on ECLS	322 (44)	97 (40)	*p* = 0.162
30 day mortality	379 (52)	119 (49)	*p* = 0.265
Death after ECLS (sec. Failure)	52 (7)	32 (13)	***p*** **= 0.003**
Vascular complications	40 (5)	13 (5)	*p* = 0.5

v-v: veno-venous support, v-a veno-arterial support

Mortality rates did not change significantly over the years, however we observed a trend towards better outcomes in the most recent patients when compared to the initial experience. We performed univariate and multivariate risk factor analysis to identify risk factors for mortality,. Multivariate risk analysis revealed Influenza (H1N1), Peak Insp. Pressure at implantation, pO2/FiO2 ratio and ECMO Score (SAVE/RESP) as well as ECLS support time as independent risk factors for mortality (See Table 4).

**Table 4:** Multivariate Risk analysis

Variable	P-value	OR	95% CI	
Influenza (H1N1)	0,004	2626	1350	5110
Peak Insp. Pressure, cmH2O	0,010	1018	1004	1033
pO2/FiO2	0,014	0,993	0,987	0,999
ECMO Score (SAVE/RESP)	0,000	0,871	0,840	0,903
ECLS running time	0,000	0,940	0,919	0,961

Multivariate

Sex Influenca PHT @LE dcm PPM PeakInsp.PressurecmH2O pO2FiO2 ECMOScoreSAVERESP

## Discussion

Various reports on ECLS supported transport have been published lately [5–9]. To our knowledge, we present one of the largest study cohorts for ECLS supported transport in this article. Outcome of our patients is in line with the mortality rates described in the literature [10]. However, the 30-day mortality rate remains unsatisfactory, especially against the background that no significant change in mortality has been seen over time. The mortality of patients supported with VA-ECMO for cardiac failure in the literature remains high with only 40 to 50% of patients surviving to hospital discharge [8, 9]. The Prognosis is even worse after cardiac arrest and cardiopulmonary resuscitation (CPR) before initiation of ECLS [11, 12].

The underlying cause for cardiogenic shock seems to depict the outcome; e.g. patients with more reversible causes of myocardial injury (eg: myocarditis, primary graft failure) have better survival than patients with post-cardiotomy in the literature. However, in our experience, post-cardiotomy was not an independent risk factor for mortality.

Our study cohort represents severely ill patients with very low SAVE and RESP scores, predicting an overall survival of around 35–45%, respectively [13, 14]. Outcome was better than predicted by the scores but still unsatisfying. Our study shows that - despite significant differences in the patient population - the outcome is similar between the in-house and transportation group. Especially with regard to the admission to ECLS times, without significant differences between in-house and external ECLS implantations, the colleagues in the secondary and primary centers are well aware of the availability of ECLS support and will consider it early on in severely ill patients. This is only possible due to a close and ongoing collaboration with the referring hospitals.

ECLS is a resource-intense, complex, interprofessional undertaking with many potentially serious complications [9, 10]. Our complication rate is low, and there is no transportation-related risk. Surprisingly, there was no significant difference in the outcome between v-a and v-v support, despite the lower predicted mortality of the v-v patients (RESP score).

Offering this support as a large center with all the optimal care options at our clinic (Transplant, VAD Therapy, surgical options) relieves the referring centers and can limit the risk of ECLS therapy as a “dead end”. In fact 7% of all patients after ECLS support were bridge-to-VAD or -transplant, offering a solution to otherwise fatal organ failure.

However, we believe that at least v-a ECLS therapy should be limited to centers with cardiovascular experience, otherwise vascular complications cannot be handled adequately. Almost 5% of the v-a patients suffered from vascular complications which otherwise could not be dealt with, despite best efforts with mandatory distal perfusion [15]. Therefore, we conclude that it is safer to offer this therapy as a tertiary center supporting the referring hospitals instead of referring centers implementing the ECLS therapy themselves. However, this requires a true 24 h/7d standby availability at the tertiary center with adequate response times.

In the past, the goal of ECLS support in patients with respiratory failure was to offer adequate oxygenation only. However, since studies revealed that mortality in these patients is mostly caused iatrogenicly by injury of the ventilation itself, the main goal is now to reduce excess peak pressure and to limit the iatrogenic stress caused by ventilation. Since the introduction of the concept of “awake ECLS”, we know that extubation of the patient is a realistic goal. Given the percentage of awake ECLS even in the transport group, avoiding the intubation of the patient seems feasible. Especially for v-a support, intubation of the patient prior to ECLS implantation can lead to severe hemodynamical distress, ultimately with the risk of subsequent cardiopulmonary resuscitation [16, 17].

Some of the v-v systems were placed with the wrong indications, e.g. v-v support for ventricular septal defects following ischemia. These real world findings are somehow related to inadequate pre-implantation diagnostics and can be a cause for major complications. However, the diagnostic options in primary and secondary hospitals can be rudimentary and therefore a misleading diagnosis can be made easily. ‘Keeping it simple’ is a rule for transport ECLS. We therefore try to avoid complicated v-a-v, v-v-a systems at outside hospitals. Instead, we rely on implantation of v-a-v or v-v-a systems at our center after initial stabilization with, in the case of both respiratory and cardiac failure, v-a- support, which was necessary in 9% of cases compared to 3% of cases in the home group.

### Limitations

One limitation of this study is the retrospective approach, however in contrary to highly selected study populations; our patients represent a real world experience. We were also not able to retrospectively include patients referred to ECLS implantation with fatal complications before ECLS implantation on site due to lack of data. Our study did not attempt to analyze the post-hospitalization outcomes and does not include a follow-up. We acknowledge that an extensive analysis of the post-hospitalization period and follow-up are mandatory to define the real mid- and long-term benefits in our patients. The lack of a control group without ECLS support is acknowledged however would be very difficult to obtain.

## Conclusion

Mobile ECLS support is a tremendous challenge. However, it is justified to offer 24 h/7d ECLS standby for secondary and primary hospitals as a tertiary center Increasing indications and total numbers for ECLS support raises the need for further studies to evaluate outcome in these patients.

## Data Availability

The datasets used and/or analyzed during the current study are available from the corresponding author on reasonable request.

## Abbreviations

ASD: Atrial septum defect
ARDS: Acute Respiratory distress syndrome
BMI: Body mass index
CPR: cardiopulmonary resuscitation
CCS: Canadian Cardiovascular Society
DLTX: Double lung transplantation
DCM: Dilative cardiomyopathy
ECLS: Extracorporeal life support
NYHA: New York Heart Association
MI: Myocardial infarction
PMR: Papillary muscle rupture
VSD: Ventricular septum defect
VAD: Ventricular assist device
